# Extended PKM Fixturing System for Micro-Positioning and Vibration Rejection in Machining Application

**DOI:** 10.3390/s21227739

**Published:** 2021-11-20

**Authors:** Francesco Aggogeri, Nicola Pellegrini, Franco Luis Tagliani

**Affiliations:** Department of Mechanical and Industrial Engineering, University of Brescia, Via Branze, 38, 25123 Brescia, Italy; francesco.aggogeri@unibs.it (F.A.); f.tagliani006@unibs.it (F.L.T.)

**Keywords:** parallel kinematic machines, flexure-based mechanism, piezoelectric actuator, vibration rejection, set-point following

## Abstract

The paper aims to present a mechatronic device able to micro-position the workpiece and to reject disturbances due to machining operation. A decoupling method is proposed for a parallel kinematic machine (PKM) fixturing platform composed by a 3-DoF flexure-based piezo-actuated mechanism. The parallel platform, with a vertical motion and two rotations, is described and its kinematics and dynamics are studied. The coupling undesirable effect is investigated based on a set of poses. To improve the quasi-static regulator model for a set-point following system, a bump less switching controller and a fine-tuning procedure, to estimate the parameter uncertainty and enable the external disturbance containment in an extended broadband frequency range, are presented. The platform and the piezo-actuator controllers are modelled based on a gain scheduling, standard ISA form method, to guarantee the stability. The accuracy is demonstrated through a set of simulations and experimental comparisons. A sensitivity analysis that evaluates the tracking performance and the disturbance rejection based on the number of signal amplitudes, frequencies, and phases is discussed. A validation phase has shown that the developed architecture presents a steady state error lower than 1.2 µm, a vibration reduction of 96% at 1130 Hz with a maximum resolving time of 6.60 ms.

## 1. Introduction

High-precision machining requires significant contributions to limit inertial disturbances and to reduce vibrational problems. To satisfy the industrial demand, several mechanisms have been developed with two main different configurations: parallel kinematic machines (PKMs) and serial kinematic machines (SKMs). Due to the reduction of moving masses, system compactness, increased load capacity, and higher stiffness, PKMs present an enhanced solution for a prescribed level of accuracy and dexterity.

A parallel architecture is a closed loop mechanism based on a moving frame linked to a static frame by a set of kinematics modules or legs [1]. However, the closed loop architecture and the kinematic constraints limit the workspace of PKMs; defined as the set of poses obtained by the joints’ configurations.

In the literature, these limitations are studied to satisfy the high-precision machining requirements with the focus on fixturing elements. Industrial awareness estimates that 40% of rejected products are associated to the fixture design [2], proving its importance. Consequently, the scientific community proposed several strategies and procedures to evaluate the performance of a fixture based on motion accuracy, workpiece-stability, workpiece-deformation, low set up time, and system adaptability [3,4].

A fixture is a mechatronic system applied to guarantee an accurate workpiece location within the workspace, rigidly holding and supporting it to the machining loads, influencing its static and dynamic performance. Fixtures can be classified into three groups: locators, supports and clampers [5]. These are detailed as follows:

- Locators are devoted to correctly positioning the workpiece in 3D space;
- Supports enable the workpiece-static load-reaction without any location or positioning function;
- Clampers provide the force to maintain the workpiece position and orientation in their initial setting avoiding the time-dependent deformation or distortion [6,7,8];
- In addition, the closed loop fixtures perform two important functions:
- Workpiece location: to locate and place the part in the workspace;
- Disturbance suppression: to maintain the workpiece in a predefined position and reject any manufacturing forces or external perturbation;

Vibrations in industrial machining applications are generated by internal and external disturbances, with a significant effect on equipment reliability and product quality. Moreover, the disturbance presence and the high-magnitude oscillation of the workpiece may result in its damage and breakage. Piezoelectric elements (PZT) have become an attractive solution for vibration reduction due to their significant electro-mechanical connection, the high-frequency response, and superior durability [9,10]. In order to reduce the existing vibrations of a precision machining with PZT modules, many control approaches are recommended to address the limitations produced by undefined dynamics, complex boundary settings, non-linear configuration, and the electrical saturation of the actuators [11].

Recent research focused on the use of flexure-based mechanisms. These elements have several advantages as negligible friction, minor backlash, and no-hysteresis [11]. Consequently, flexure-based devices driven by PZT-actuators represent the optimal arrangement for high-precision machining [11,12], as such actuators can generate continuous extension and backward movements with high-resolution, high-stiffness, and high-driving force within an extended-response range.

Several control approaches including robust control, adaptive control and neural network control have been developed and investigated [13,14,15] to improve the dynamic performance and managing the PZT non-linearities. Authors concentrate the proposed research in thin-wall machining applications. The literature presents different solutions to apply actuators and control strategies to contain the vibrations in thin-walled machining [16,17,18]. Active dampers are one of the main solutions applied for reduce vibration contribution in variable machining conditions. In this way, an interesting study was proposed by Yang et al. [19] that designed an active and lightweight device based on eddy current damping (EDC) to attenuate the vibrations produced during the machining of a thin-walled aluminum frame. Based on the electromagnetic induction, the authors achieved a reduction of the machining vibrations of up to 84%. An alternative approach was presented by Wang et al. [20] with an active milling vibration control system based on a time-space varying proportional derivative (PD) which varies the regulator parameters according to the position of the milling point. The system used a piezoelectric patch as an actuator to suppress the vibration of the thin-wall workpiece milling. Despite obtaining higher control capabilities with respect to the standard PD, the simulation and validation was carried out for a known path with a rectangular thin-wall workpiece. An alternative variable PD controller was implemented in [21] with a fuzzy inference model based on the ANFIS system, proving its capabilities for thin-walled milling parts model. Furthermore, this kind of application is based on the flexible beam active vibration control basis [22,23,24]. The main issues are related to the actuator positioning (workpiece dependent) and the limitation upon the machining of the corresponding surface.

In order to overcome these problems, the active holders are studied, as in Diez et al. [25], where the authors employed a compliant piezo-actuated flexure to compensate the deformations of the workpiece, improving the final dimensional error from 246.0 µm to 36.0 µm. The compensation system considered the static deformations experienced in machining comprising of low-rigidity parts. The control system is based on a reference model that runs in real time which is a good procedure to compensate for dimensional errors due to part deformation in milling machining. A similar unidirectional controlled structure was presented in [26] based on a piezo stack actuator and a property support base designed with proper flexure hinges to allow the compensation of the workpiece machining disturbances. Despite the good results, the control system was based on an analytical model to estimate the force and consequently the workpiece is dependent. Moreover, the dynamometer requested as feedback for the regulator tends to be difficult to install and limits the dimensions of the machinable workpieces. However, the authors managed to obtain an industrially appliable device by creating the corresponding supports upon the given sensor. Further configurations were based on a two-directional active piezo-based workpiece holder as the flexure-based system in [27] where the authors employed a linear motor for the large motion and a piezoelectric actuator for the fine stage positioning obtaining a reduction in the maximum tracking error of 83% for a sinusoidal profile machining test. Similarly, in [28,29,30] the authors presented different piezoelectric based moveable frame obtained through flexure hinges and springs. An adaptive application based on the filtered-X LMS algorithm was applied by Rashid and Nicolescu [31] to improve the dynamics of the fixture system by eliminating the vibration signal generated by the cutting process, succeeding in improving surface quality and tool life. The study reports a test with simple geometry affected by a forces profile lower than 800 N amplitude, with a frequency range under investigation lower than 250 Hz. The campaign outcomes were satisfying with a Rz lower than 3.8 µm. A different holder was implemented in [32] for variations of ±50 μm and forces up to 2500 N, the control was based on a relative positioning error between the tool and the machine table. To improve the system capabilities, an optimal LQG (linear quadratic gaussian) controller was implemented by Parus et al. [33] for an active clamping system based on piezoelectric actuators. The system suppresses the vibrations through the forces acting on the workpiece table independently of the vibration nature and level. An LQG controller was employed with a Kalman filter in [34] to actively introduce damping into the system with a high-voltage piezoelectric actuator, equipped with a thermal management system required for long-term operations, and a laser sensor. The results showed an increment capability in the depth of cut from 0.6 mm up to 2.3 mm for a spindle speed of 240 RPM. In a similar approach, in [35] the authors employed a laser sensor for a runout control based on a piezoelectric actuator. The sensor chosen for the feedback signal, a laser vibrometer, despite providing an accurate vibration measurement, it has several disadvantages based on the measurement method, for example, it cannot be used when a coolant is applied. Differently, an artificial neural network-based controller was implemented in [36], that was used to determine the chatter frequencies and remaining vibration amplitudes that were further optimized by a genetic algorithm approach. Nevertheless, the approach requires an initial training phase and optimization.

Although the literature presents various vibration control methods and significant achievements are demonstrated, further studies are required to investigate the vibration compensation in broadband frequency range in combination with precise workpiece positioning.

**Figure 1:**
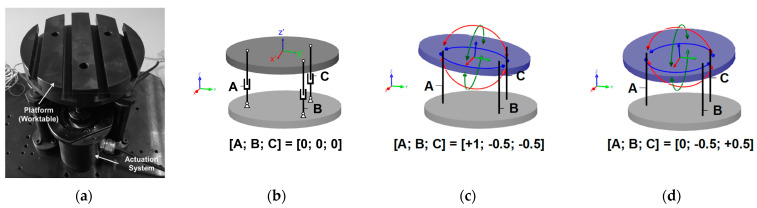
(**a**) PKM locator and support prototype [7]; (**b**) kinematic structure of a 3-DOF PKM; (**c**) view of pose rotation around *X*-axis; (**d**) view of pose rotation around *Y*-axis.

The main contribution of this paper is to extend and validate a compact fixture system that regulates in real-time a set of piezo-actuated modules in an industrial environment. Authors propose a platform based on a 3-DoF (degrees of freedom) structure, established on the Kutzbach Grubler equation as described in [8], driven by preloaded piezo-stack actuators that act on the vertical axis at different table points. Figure 1a presents the developed prototype, in Figure 1b the schematic diagram of the active platform is shown. Figure 1c,d show two different poses within the tolerable workspace.

**Figure 2:**
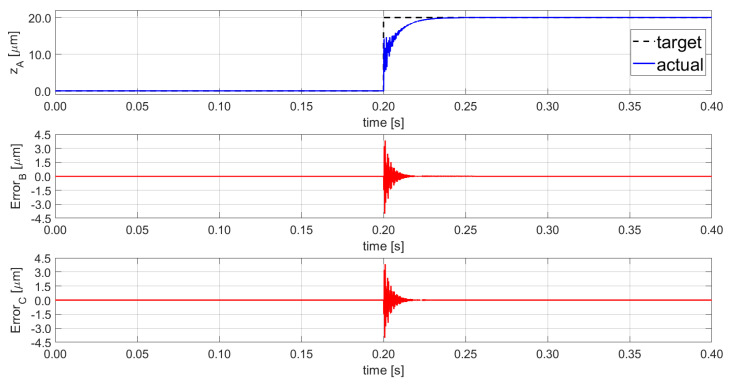
Step function following performance of PKM platform—PZT module A and the PKM coupling effects, the errors observed on PZT-module B and PZT-module C.

The platform’s coupling effect is generated by the actuated leg that exerts a disturbance on the other legs through the moving frame motion. The effect can be investigated with a sequence of pose tests based on step signals as target input. The experimental campaign results of the original platform on a given commercial machine tool [8] are shown in Figure 2. A step input is given on module A, and the corresponding coupling is measured for modules B and C. In the prior work, the scope was limited to clamping recovery (low-frequency disturbances) and the implemented regulator was developed for a quasi-static application. The actual coupling effect presented in Figure 2 for a 20 µm input on module A causes a peak deviation of ±4.02 μm for both B and C modules. This undesirable effect is a main challenge that needs to be solved to allow the vibration rejection function for the micro-positioning platform.

The additional contribution of this paper is to progress the design developed in [7] by implementing a dynamic regulator to allow disturbance rejection capabilities and to contain the flexure-based coupling effects in thin-plate precise machining. This is achieved by a suitable selection of the control algorithm and a gain-scheduling approach. The proposed method uses a bumpless switching controller and a fine-tuning procedure to estimate the parameter uncertainty and enable the external disturbance containment in an extended broad-band frequency range. The platform and the actuator controllers are designed based on a gain scheduling PID (proportional integral derivative), in standard ISA form, to guarantee the stability [37,38,39,40,41]. The accuracy is proved through a set of simulations and experimental comparisons, performed on a proposed model, for the two objective functions: set-point tracking and disturbance rejection.

## 2. The Concept and Formulation of 3-DoF PKM Piezo-Actuated Platform: Kinematics and Dynamics

**Figure 3:**
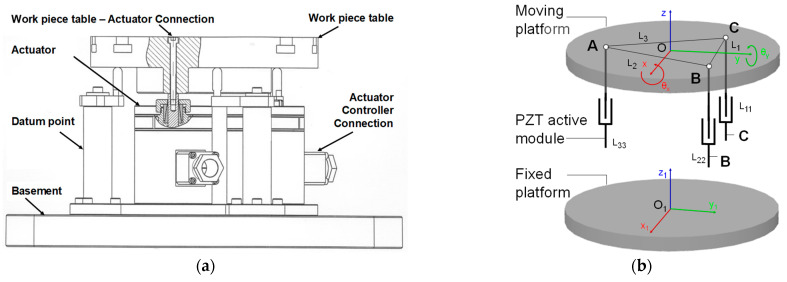
(**a**) Geometric representation of 3-DoF PKM assembly simplified model; (**b**) [*z*; *θ_x_*; *θ_y_*] motions of the PKM platform by super-position of active modules.

In a three-dimensional workspace, a rigid body requires the support of 3 constrained points to guarantee the location’s definition; this postulate is the base of the 3-DoF parallel mechanism design. The proposed active PKM system, shown in Figure 3a, is composed of a top moving table, a fixed base platform, three (prismatic joint-spherical joint-prismatic joint) PSP active legs r_i_ (i = 1, 2, 3) and three datum points for supporting pillars with no actuation. Each PZT-module, installed between the basement and the workpiece table, contains a piezoelectric controlled actuator that provides proper forces, damping capacity, and high stiffness. Due to its electromechanical property, the piezoelectric elements are affected by hysteresis, and this may be minimized by using an inner closed loop regulator, which can compensate the parasitic error in the active mechanism. In order to overcome these motion problems, three precision capacitive sensors are used in the table to measure the contribution of each actuator and the moving platform, forming the final closed loop system [11,42,43]. The PZT-actuator selected for this active fixture platform is the PST 1000/25/40 VS35, which has a maximum stroke capability of 40 µm, it has an axial stiffness of 450 N/µm, and a blocking force of 25.0 kN. The piezoelectric modules are fixed on the base frame and placed on a circumference, at equal angular distance between each other, around the center of the moving frame; and are attached to the latter through a rigid connector. The PKM platform is represented by a MIMO (multiple input multiple output) system or it could be synthetized by three parallel SISO (single input single output) subsystems. The moving frame can move forward-backward (maximum 40.0 μm stroke) on a torsional-flexure mechanism in accordance to a digital input signal along the vertical-axis (compliant direction) for the SISO module. The selected high-precision capacitive sensor has a resolution of 7.3 nm considering an operative range of 100.0 μm with a response-band of 0–17.5 kHz. A PZT-amplifier controlled by a digital computer board (dSPACE-1104), with an output peak voltage of 5.0 V, is adopted to generate the control voltage for the piezoelectric actuator motions, and the piezoelectric amplifier component corresponds to the SVR1500, with an amplification factor of 100.0 ± 0.1. Finally, a personal computer is used to run the MATLAB-Simulink control, the GUI user-interface, and to supervise the control functions.

To describe the platform motion, two coordinate frames are defined; one local O-XYZ is positioned on the moving frame and one global O_1_-X_1_Y_1_Z_1_ is fixed on the ground, as shown in Figure 3b. The coordinate frames are aligned along the *z*-axis and present the same orientation when the moving workpiece table is in its initial position.

The control presented in this paper acts on [*z*; *θ_x_*; *θ_y_*] directions which are defined with respect to the center-of-mass of the workpiece table. The direction z represents the vertical movement aligned to the *z*-axis, *θ_x_* is the rotation about the *x*-axis, and *θ_y_* is the rotation about the *y*-axis. Considering the moving platform as a rigid element, the motion parameters ([*z*; *θ_x_*; *θ_y_*]) can be allocated among points A, B, and C, which compose an equilateral triangle (ΔABC) on the mobile platform with side length L. Point O corresponds to the center of the circumscribed circle of ΔABC. The *y*-axis is positioned at the middle of the CB segment between the two passive spherical joints; moreover, the *z*-axis corresponds to the normal vector of the top moving frame; finally, the *x*-axis can be identified through the right-hand rule. The vertical and tilt-tip motions of the reference point O can be obtained through the following Equations (1)–(3):

(1) $$O=J_{0}\;\cdot \;I={\left[\begin{array}{ccc} z & \theta_{x} & \theta_{y} \end{array}\right]}^{T}$$

(2) $$I={\left[\begin{array}{ccc} z_{A} & z_{B} & z_{C} \end{array}\right]}^{T}$$

(3) $$J_{0}=\left[\begin{array}{ccc} \frac{1}{3} & \frac{1}{3} & \frac{1}{3}\\ \frac{2}{3\cdot R} & -\frac{1}{3\cdot R} & -\frac{1}{3\cdot R}\\ 0 & -\frac{\sqrt{3}}{3\cdot R} & \frac{\sqrt{3}}{3\cdot R} \end{array}\right]$$

where I is the input vector, corresponding to the displacements of the three PZTs; and **J_0_** is the transformation matrix from I to O, which is a function of the circumference containing ΔABC radius (R).

**Figure 4:**
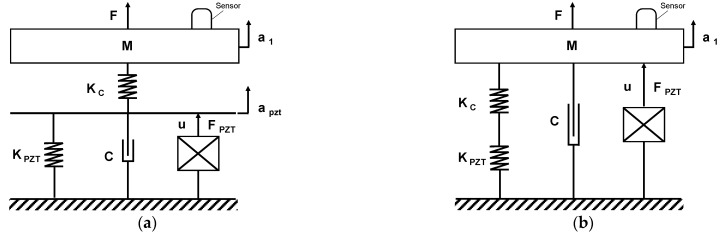
(**a**) Flexure-based mechanism with actuator connection scheme for a single axis; (**b**) simplified model of the PZT platform scheme for a single axis.

The dynamic differential equation of the active piezoelectric platform motion in Figure 4 can be described, using the Newton’s second law of motion, by the following Equation (4):

(4) $$M\ddot{d}\left(t\right)+C\dot{d}\left(t\right)+Kd\left(t\right)=F\left(t\right)+BV\left(t\right)$$

where ***M***, ***C***, ***K***, and d correspond to the mass matrix, the modal damping matrix, the stiffness matrix and the displacement vector, respectively; the factor *F* describes the resultant external forces applied to the corresponding rigid-mass; *B* is the force/voltage transformation; and *V* corresponds to the input voltage of PZT module. The model parameters ***M***, ***C***, and ***K*** were analytically estimated with the finite element (FE) analysis, as presented in [7]. The ground frame position is denoted as $a_{0}$ = [*z_0_*; *θ_x_*_,0_; *θ_y_*_,0_], the moveable frame position as $a_{1}$ = [*z*_1_; *θ_x_*_,1_; *θ_y_*_, 1_], and the actuator voltages as u = [*V*_A_; *V*_B_; *V*_C_]. These coordinates are chosen based on the vertical actuator forces and the vertical measurements configuration, as represented in Equation (5):

(5) $$u\left(t\right)=B\left[\begin{array}{c} V_{A}\left(t\right)\\ V_{B}\left(t\right)\\ V_{C}\left(t\right) \end{array}\right],\;a_{0}\left(t\right)=R_{0}\left[\begin{array}{c} a_{0,1}\left(t\right)\\ a_{0,2}\left(t\right)\\ a_{0,3}\left(t\right) \end{array}\right],\;a_{1}\left(t\right)=R_{1}\left[\begin{array}{c} a_{1,1}\left(t\right)\\ a_{1,2}\left(t\right)\\ a_{1,3}\left(t\right) \end{array}\right]$$

In Equation (5), *a_0,j_* and *a*_1*,j*_, denote, respectively, the ground frame’s and moving platform’s positions, where j corresponds to the *j_th_* PZT element. Moreover, the sensors assessing *a*_0,1_, *a*_0,2_, and *a*_0,3_ are intended as perfectly collocated. Finally, the actuators forces are stated by *u_j_*, and **B, R_0_, R_1_** in ℝ^3×3^ represent the static transformation matrices.

The initial structure’s model is presented in Figure 4a, where the connection mechanism is denoted by the K_c_ stiffness positioned upon the PZT actuator (K_pzt_, C, F_pzt_). The chosen scheme created an internal displacement variation caused by the stiffness in series to the PZT model, which increased the complexity of the final system. Considering that K_c_ was high, the model was simplified into the second scheme shown in Figure 4b, where the actual stiffness of the system is kept unchanged but the inner displacement, represented by *a_pzt_* in Figure 4a, is neglected.

**Figure 5:**
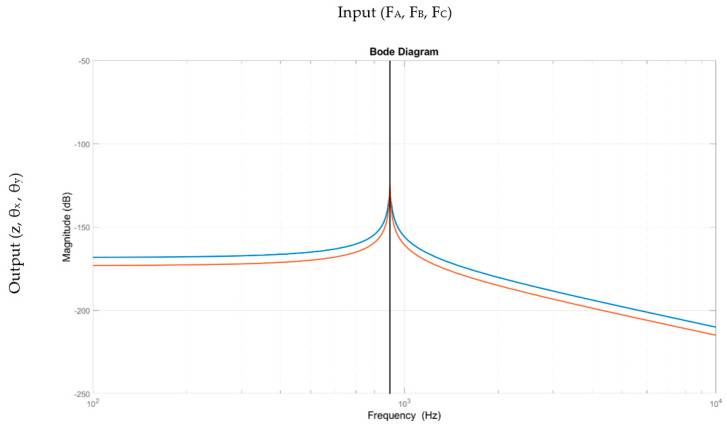
Bode diagram of platform modal comparison, blue line—K_pzt_ and K_c_ in series-wise setting and red line—flexure-based mechanism with interface node for actuator connection.

In order to validate the simplified model, a modal comparison scheme was performed, by evaluating the input forces (A, B, and C channels) for each active module with the corresponding displacements ([*z*; *θ_x_*; *θ_y_*]). The results are summarized in Figure 5, where a single SISO model outcome is shown, representative of all the SISO tests. The difference between the architecture with interface node (Figure 4a) and the simplified scheme (Figure 4b) is lower than 4.93 dB. The results confirm the implementation of the second scheme without affecting the overall performance. Nevertheless, this simplification error was taken into account when the control tuning was carried out, by implementing a final regulator with higher robustness. Finally, it is worth mentioning that based on the specific reference frame selected, the *θ_y_* rotation is null for the input force A as it is positioned along the y axis, as shown in Figure 3b.

### The Modelling of the Piezo Actuator

The piezoelectric stack actuator model is described by a linear system, as stated in Equations (6) and (7). The hysteresis effect is neglected, and the polarization direction is selected along the body stack in vertical axis [44,45]. The piezoelectric stack actuator can be written as:

(6) $$S_{3}=d_{33}E_{3}+s_{33}^{E}T_{3}$$

(7) $$D_{3}=\epsilon_{33}^{T}E_{3}+d_{33}T_{3}$$

where *S*_3_ is the mechanical strain, *D*_3_ is the electric displacement, *E*_3_ is the electric field, *T*_3_ is the mechanical stress, *d*_33_ is the piezoelectric constant, *s*^E^_33_ is the elastic compliance at constant field *E*, and ϵ^*T*^_33_ corresponds to the permittivity at constant stress *T*. According to the assumption that each layer has a thickness *h*, a cross-sectional area *A*, and the electrical potential between the two phases of each layer is *dV*, the following equations are obtained:

(8) $$S_{3}=\frac{\Delta h}{h},E_{3}=\frac{dV}{h},T_{3}=-\frac{F}{A}$$

Furthermore, as in [46,47] the displacement of each layer, Δh, can be formulated as:

(9) $$\Delta h=-\frac{h\;s_{33}^{E}\;F}{A}+d_{33}dV$$

Considering a PZT with *n* layers, with identical polarized direction and stack form, the resulting final displacement, ΔL, can be computed as:

(10) $$\Delta L=n\Delta h=-\frac{\left(n\;h\right)s_{33}^{E}F}{A}+d_{33}\left(n\;dV\right)=-\frac{F}{K_{m}}+d_{33}V$$

where $\frac{A}{\left(n\;\cdot h\right)\;\cdot s_{33}^{E}}$ is the equivalent stiffness, denoted as *K_m_*. Finally, in order to obtain the voltage-force function, Equation (11) is rewritten as:

(11) $$F_{PZT}=-K_{m}\Delta L+K_{m}d_{33}V$$

## 3. Control Algorithm Design and Methodology

This section is devoted to the presentation of the control design methodology. The controller is implemented for the MIMO system, employing the collocated measured position signal as feedback input for each axis. The desired controller should increase the robustness during disturbance rejection and expand the set-point following the frequency range of the PZT platform. In addition, two different sets of tuning parameters have been discussed. The procedure consists of two main phases:

- The design of a precise set-point following controller and definition of a disturbance rejection strategy;
- Bumpless switching application and controller tuning.

### 3.1. Controller Design

The regulator synthesis is based on a linear dynamic model that connects the platform position to the PZT voltage. A PID controller was preferred for the investigated thin-wall application, defined in standard ISA form as follows:

(12) $$u_{PID}=K_{p}\left(\left(bSP-Y\right)+\frac{T_{d}s}{1+\frac{T_{d}s}{N}}\left(cSP-Y\right)+\frac{1}{T_{i}s}\left(SP-Y\right)\right)$$

where *SP* is the set-point signal, *Y* is the measured output, *K_p_* is the proportional gain, *T_i_* is the integral time constant, *T_d_* is the derivative time constant, *b* is the set-point weight, and *c* is the derivative weight.

A back-calculation scheme is provided introducing an add-on parameter, the tracking time constant (*T_t_*), that is obtained as $T_{t}=\sqrt{T_{i}\cdot T_{d}}$. The *T_t_* factor supports the saturation avoidance by regulating the integral action of the inner loop. After the set-point reaching, the controller parameters are re-tuned to enable the disturbance rejection. These disturbances are both external, with variable contribution due to the machining process itself, and internal, generated by the coupling effect between the actuators.

The chosen approach manages to handle and limit the inner coupling issues between the three actuators, and it allows the consideration of the MIMO system as three SISO subsystems. Furthermore, a gain-scheduling scheme is deployed to achieve both set-point following, and the disturbance rejection functions. During the initial positioning phase the platform is affected by limited disturbances, with static or low-frequency contributions as the interaction between tool and workpiece is still null. Once the cutting tool reaches the workpiece higher disturbances appear, whose frequency content depend on the vibration’s source. A bumpless switching is implemented to switch the controller to the disturbance rejection scheme, by retuning the regulator.

### 3.2. Controller Tuning

The resulting SISO transfer function has two complex pair of poles and one complex pair of zeros, as follows:

(13) $$G\left(s\right)=K_{p}\cdot \frac{\left(s^{2}+2\xi_{z}\omega_{z}\cdot s+\omega_{z}^{2}\right)}{\left(s^{2}+2\xi_{p_{1}}\omega_{p_{1}}\cdot s+\omega_{p_{1}}^{2}\right)\cdot \left(s^{2}+2\xi_{p_{2}}\omega_{p_{2}}\cdot s+\omega_{p_{2}}^{2}\right)}$$

**Figure 6:**
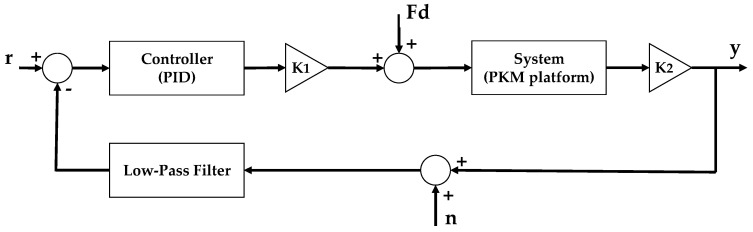
SISO closed loop representation.

The transfer function presented in Equation (13) links the force to the output displacement of the platform. Therefore, the PZT transfer function that computes the actuator force from the input voltage is required ($K_{1}=d_{33}\cdot K_{pzt}$), it is worth noticing that the force limitation given by the stiffness behavior of the actuator, as presented in Equation (11), is considered within the system transfer function ($K_{pzt}\cdot \;X_{pzt}$). Moreover, a gain corresponding to the displacement conversion factor ($K_{2}=10^{6}$) is implemented. Finally, a low-pass filter is used to reduce the measurement noise (*n*) on the feedback line. The closed loop scheme is shown in Figure 6, where *Fd* corresponds to the disturbance force, and *r* is the reference signal.

An analytical tuning procedure was implemented in order to contain the system resonance frequencies. The free remaining parameter was *K_p_* which was set based on the resulting control margins and the actuator saturation. During the set-point following phase, *K_p_* was tuned robustly to avoid any overshoot, and it limits the dynamic oscillation of the PZT and the resulting inertial disturbances. Differently, the disturbance rejection scheme required a more aggressive tuning in order to contain the machining disturbances. The corresponding value of *K_p_* was higher as it can be observed in Table 1, where the final tuning values for the controller parameters are presented.

**Figure 7:**
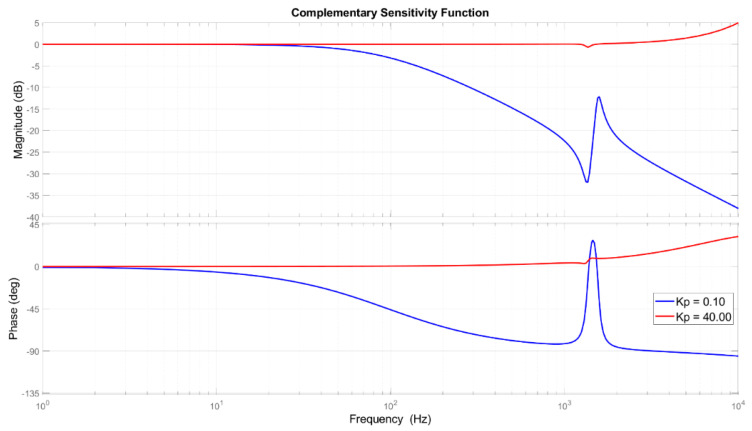
Bode diagram of the complementary sensitivity function.

**Figure 8:**
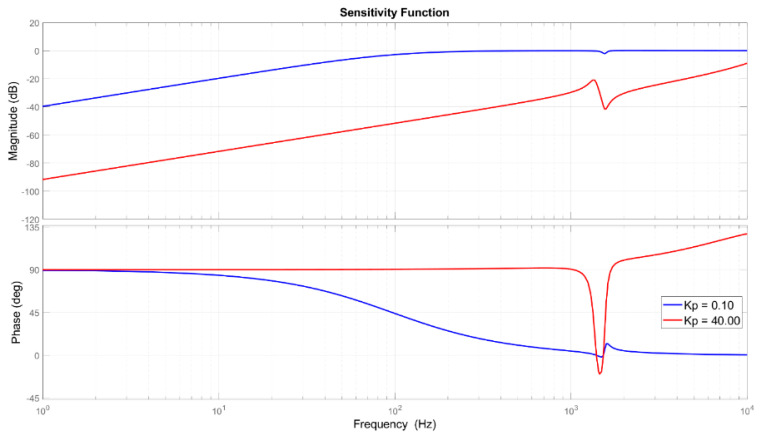
Bode diagram of the sensitivity function.

The tuned parameters were found based on two main functions, the complementary sensitivity function (shown in Figure 7), which is the ratio between the output signal Y(s) and the input signal R(s), and the sensitivity function (presented in Figure 8), which relates the output signal Y(s) with the disturbance signal Fd(s). Instead of using the actual force disturbance signal, the corresponding displacement disturbance ($F_{d}\cdot G\cdot K_{2}$) was used to correctly evaluate the amplitude containment of the controller.

The complementary sensitivity function, shown in Figure 7, presents the system capability to follow a given set-point in input with a certain dynamic behavior given by the cut-off frequency (frequency at which the line crosses the 0 dB axis). Moreover, higher speed response will result in higher power consumption. This trade-off in combination with the overshoot avoidance were used to determine the *K_p_* for the set-point following phase. The hypothesis is to use quasi-static input reference signals.

Finally, to determine the disturbance rejection gain parameter the sensitivity function was used, as shown in Figure 8 where it can be observed that the disturbance rejection tuning contains a minimum of 45 dB up to 200 Hz, and a minimum of 20 dB (at the resonance point near 1050 Hz) up to 1.5 kHz.

## 4. Numerical Simulation and MIMO Validation

A set of time-domain simulation tests were carried out to verify the performance of the designed controller for the MIMO piezo-driven platform, for both set-point tracking and disturbance rejection in MATLAB-Simulink environment. The MIMO system is represented by three SISO systems, each with their own actuator, sensor, and regulator. During the validation, the platform is driven to a given steady state position by the three PZT actuators, and it performs the set-point following phase. Therefore, in order to analyze the tuning rules presented in Section 3.2, several step inputs with different amplitudes and step times have been investigated. Due to the absence of machining disturbances while the initial positioning phase is carried out, this phase was tuned accurately in order to minimize the time required to reach the final position while avoiding any overshoot. Nevertheless, the coupling effects between the lines, that were neglected during the control implementation and the corresponding SISO system simulations create inner disturbances that require further consideration. The vibration suppression is more critical with respect to the set-point following, as the request is to manage sinusoidal disturbances, with various frequencies and amplitudes, ramps, and steps. The examined frequency range is a broad band from constant disturbance up to 1500 Hz, with maximum disturbance expected during the machining process. Furthermore, to test the tuning parameters, this study evaluates the response at the minimum containment frequency, at the platform’s resonance. In addition, the phase changes of the sinewaves that determine the amount of containment of the control action are also assessed. The amplitude of the chosen sinewaves is larger than any expected disturbance that could occur during the machining process, and is thus used as a threshold reference. Additionally, to evaluate the control results, a residual error band is defined to consider the solution compliant, as ±1.5 µm. The numerical campaign is presented in Table 2, where the input signal for the three actuators and the resultant disturbance containment percentage are highlighted.

### 4.1. SISO Coupling Effect

**Figure 9:**
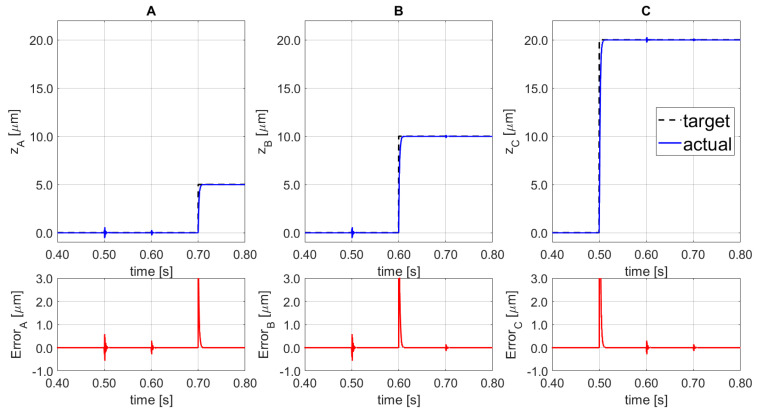
Set-point following (top) and corresponding error (bottom) for the 3 SISO (**A**–**C**).

The impacts of the coupling effect between the three SISO lines are described in Figure 9. A step-input function with multiple amplitudes is defined: first, a vertical step of 20.0 µm on SISO_C_ at 0.50 s, afterwards, a vertical step of 10.0 µm on SISO_B_ at 0.60 s, and finally, a 5.0 µm step on SISO_A_ at 0.70 s. The corresponding coupling effects on A, B, and C lines are displayed by red solid lines at the bottom figures together with the set point following error.

In comparison to the results obtained in the previous work, reported in Figure 2, the proposed control system regulates the coupling effects with a residual maximum displacement of ±0.59 µm, and it reduces the disturbance by 98% with a recovering time lower than 8.9 ms. The proposed new controller improved the final result by 85.95% when compared to the previous coupling disturbances (±4.02 μm within 17.4 ms). Moreover, the settling time is reduced by 48.90%. During all three input signals the regulators were set to the set-point following tuning, the results should be increased during the machining, as the disturbance rejection capability is improved due to the related tuned parameters.

### 4.2. Set-Point Following: Tracking Performance

**Figure 10:**
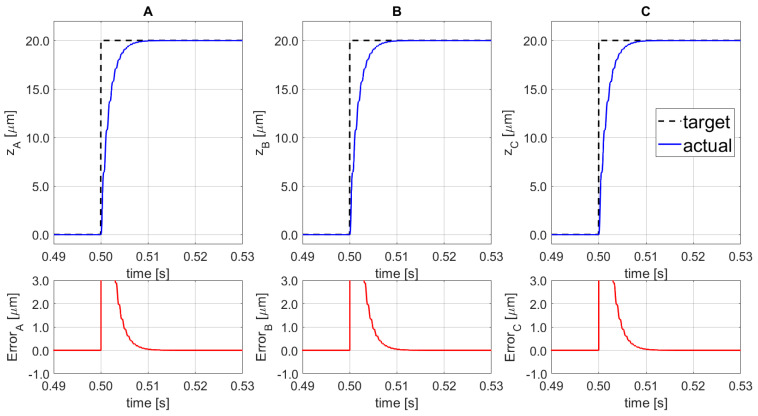
Set-point following (top) and corresponding error (bottom) for the 3 SISO (**A**–**C**).

The numerical results on the set-point following along the vertical z-direction, platform lifting without orientation variation, are presented in Figure 10. The time required to reach the 98% of the target, a 20.0 µm displacement, is equal to 6.6 ms (settling time) for the three actuation points.

**Figure 11:**
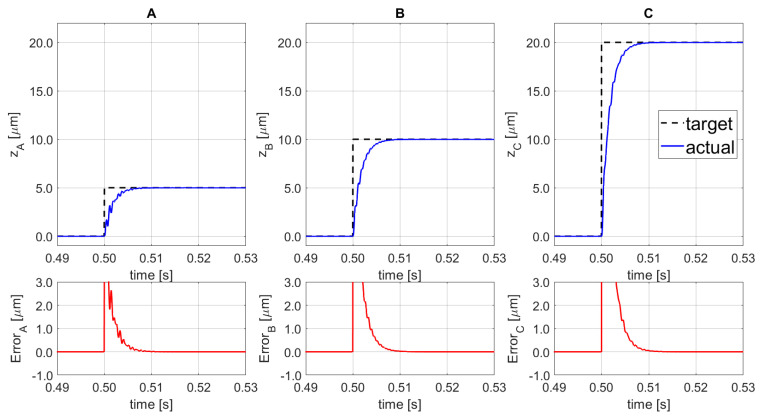
Set-point following (top) and corresponding error (bottom) for the 3 SISO (**A**–**C**).

Moreover, to demonstrate the set point following capability of the proposed system, a combination of lifting and orientation variations was implemented, as shown in Figure 11. The input signal arrives at 0.50 s, for SISO_A_ the step-displacement input amplitude is 5.0 µm, for the SISO_B_ is equal to 10.0 µm, and for SISO_C_ a set-point of 20.0 µm is applied. The final platform’s position corresponds to the following: z = +11.67 µm,$\alpha_{x}=-123.46\;\mu \mathrm{rad}$, and $\alpha_{y}=+106.92\;\mu \mathrm{rad}$. The final results show that the regulator is able to reach the reference value, without overshoot, with a settling time (at 98%) of 6.6 ms.

### 4.3. Disturbance Rejection Performance

The test validation of the disturbance rejection capability is determined by the compliance to the selected threshold of ±1.5 μm. Moreover, the simulation campaign permits to evaluate the control performance out of the allowable physical stresses in the open loop, for instance, oscillations of ±50 μm could determine the PZT actuator breakage.

**Figure 12:**
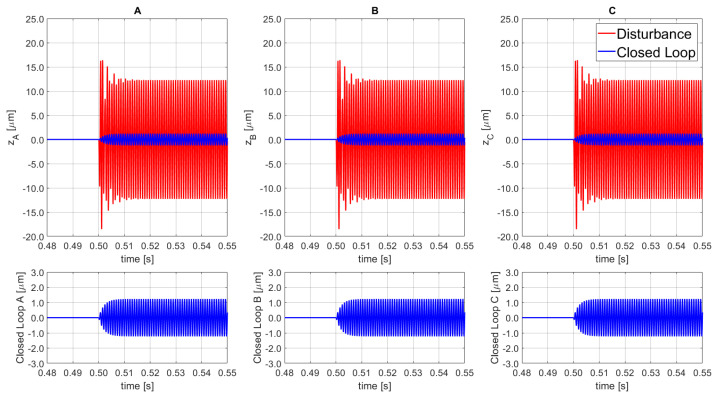
Disturbance open loop (top red, solid line) and disturbance rejection closed loop (bottom blue, solid line) for the 3 SISO (**A**–**C**).

The performance outcomes are presented in Figure 12, where the dynamic response to a vertical oscillation disturbance near to the resonance frequency of the PKM platform is presented. The disturbance signal is characterized by an amplitude of 1500 N, phase equal to 0, and a frequency of 1130 Hz for all 3 SISO actuators. Finally, the regulator is able to contain 89.98% of the disturbance amplitude in combination with the selected error band of compliance.

**Figure 13:**
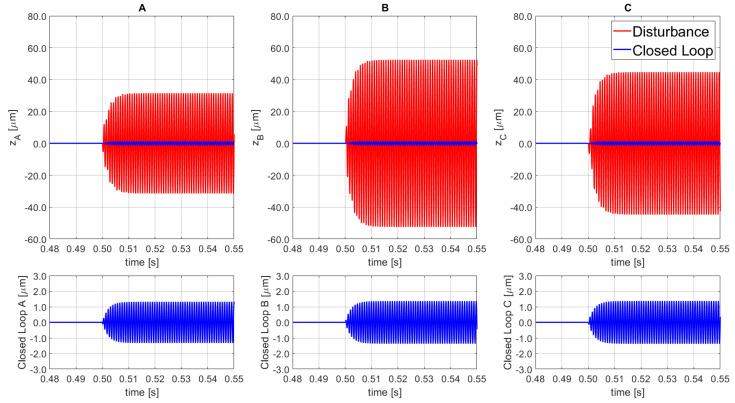
Disturbance open loop (top red, solid line) and disturbance rejection closed loop (bottom blue, solid line) for the 3 SISO (**A**–**C**).

Furthermore, a sensitivity analysis is performed to evaluate the impact of the disturbance phase variation on the control performance. Figure 13 shows the results based on a perturbation with constant amplitude of ±31.5 μm for SISO_A_, ±52.5 μm for SISO_B_ and ±44.7 μm for SISO_C_, with frequency of 1130 Hz for all three disturbances, and phase equal to π/2 for SISO_A_, π for SISO_B_, and 0 for SISO_C_. The physical meaning of the selected disturbance is an oscillation of the XY-plane about both the *x*-, and *y*-axes. As highlighted in the corresponding figure, the obtained containments for each SISO line were: 95.81% for SISO_A_, 97.41% for SISO_B_, and 96.93% for SISO_C_. Moreover, despite the high-amplitude disturbances, the final error remained within the compliance band of ±1.5 μm.

## 5. Tracking Performance under Continuously Varying Disturbances

Experimental tests are conducted to verify the developed model and to establish performance measurements for the piezo-driven flexure-based mechanism. Testing campaigns were performed on a vertical milling machine with a 2.50 mm diameter and a two-teeth tool to study the machining disturbances behaviour. The workpiece material was a thin-wall component made in high-grade carbon steel. From these experimental tests, a model for the disturbance signal was obtained. The machining disturbance signal (d) can be represented as an unknown deterministic signal with the following mathematical formulation:

(14) $$d\left(t\right)=\overset{n}{\underset{l=1}{\sum }}A_{l}\left(t\right)sin\left[\omega_{l}\left(t\right)t+\phi_{l}\left(t\right)\right]+rand\left(t\right)$$

where $A_{l}$ represents the amplitudes, $\omega_{l}$ the frequencies, and $\phi_{l}$ the phases. Finally, a random variable signal is added. For this application, it can be defined as sudden load changes that could physically represent the tool breakages or the tool first contact with the workpiece. The step disturbance signals selection of ±2000 N is due to the evaluation of edge capabilities of the developed PKM platform.

In the simulated tests, the PKM platform was arranged with a defined position and orientation determined by the following three displacement signals: SISO_A_ as 15 μm, SISO_B_ as 12.0 μm, and SISO_C_ equal to 10 μm; which in terms of platform motion corresponds to z = 12.33 µm, $\alpha_{x}=49.38\;\mu \mathrm{rad}$, and $\alpha_{y}=-21.38\;\mu \mathrm{rad}$.

**Figure 14:**
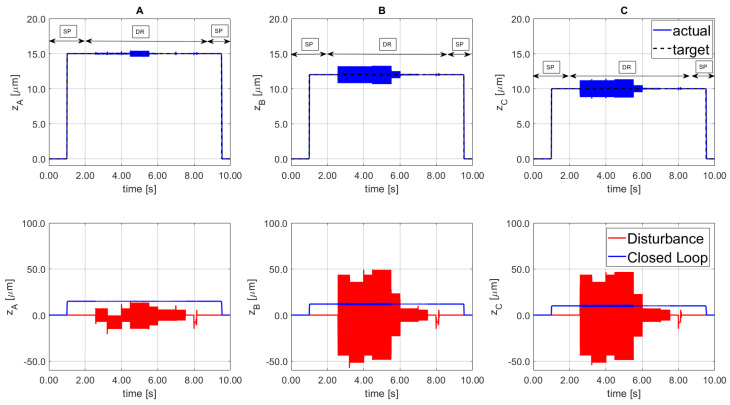
Cutting machining signals implemented in PKM platform (top). Disturbance open loop (bottom solid line) and disturbance rejection closed loop (bottom blue, solid line) for the 3 SISO (**A**–**C**).

During the initial tracking phase, the set point (SP) tuning is used. After 1.50 s a cutting machining disturbance is simulated, according to Equation (14), and thus the tuning is changed to disturbance rejection (DR). The disturbance was stopped at 8.50 s, where the SP tuning was implemented to transport the platform to the initial setting position/orientation, home position. During the 7.0 s disturbance interval, several sinewaves were implemented, from 50 Hz to 1500 Hz with various phases and amplitudes, from 375 N to over 1500 N. The results are shown in Figure 14 where the top Figures represent the tracking signals for each line.

Figure 14—bottom side, shows the disturbance signal d (solid red line) and the resulting signal from the PKM platform (solid blue line). The residual vibration remained within the threshold error specified for thin-wall machining application.

The system confirms a containment greater than the 95% of the 1.5 kN amplitude disturbances with the chosen tuning parameters. A step disturbance for both positive and negative directions is applied at 8.0 s and the system demonstrates the ability to suppress the perturbation, that in industrial setting could damage the PZT actuator. The frequency variability of the selected disturbances does not affect the regulation performance, the proposed architecture demonstrates the promising findings.

## 6. Conclusions

A 3-DoF compact parallel platform with a vertical motion and two rotations was used to demonstrate the extension of fixturing system functions as micro-positioning and vibration rejection in machining application. The PKM mechanism includes the bottom fixed frame, three piezoelectric actuators, three capacitive sensors, three datum point passive pillars, the top frame, and the flexures that perform the motions. The platform and the piezo-actuator elements were analyzed and modeled to describe the static and dynamic performance of the motion system. The controller was established for the MIMO (multiple input multiple output) system, using the collocated measured position signal as feedback input for each loop. The control structure corresponds to a PID in standard ISA form with a gain scheduling scheme. The actuator coupling effect was reduced with a maximum displacement of ±0.59 µm and a recovering time lower than 8.9 ms as response to a 20.0 µm tracking. The regulator robustness was increased during the disturbance rejection, and it has expanded the set-point following frequency range of the PZT platform, obtaining a final broadband rejection capability up to 1.5 kHz. The final parameters, tuned based on both the sensitivity and complementary sensitivity functions, obtained a final containment as 50 dB at 300 Hz and 20 dB at high frequency (1.3 kHz). To effectively verify the proposed approach, simulations and real-time experiments were carried out. The sensitivity analysis, the tracking results, and the disturbance rejection outcomes confirm the compliance of the simulation models. In the experimental case study, the signals were generated based on a model obtained from extracted data during machining and used to test the disturbance rejection platform capabilities. The tests were carried out within the platform workspace of: z = ±20 µm, α_x_ = ±427.67 µrad, and α_y_ = ±493.83 µrad. Furthermore, sudden load profiles were applied to the PKM platform with different amplitudes, from 375 N to 1500 N, variable phases and multi-tone frequencies close to critical resonances. The system confirms a containment greater than the 96% of disturbances with the chosen tuning parameters. Future work will focus on noise reduction, sensor precision, and measurement techniques to improve the robustness of the proposed feedback control approach.

## Figures and Tables

**Table 1:** Final controller parameters.

Controller	*K_p_*	*T_i_*	*T_d_*	*N*	*T_t_*	*b*	*c*
Set Point Following	0.1	1.6190 × 10^−5^	0.0012	768.4436	1.3938 × 10^−4^	0.8	0.0
Disturbance Rejection	40.0					1.0	1.0

**Table 2:** Disturbance rejection: simulation and results.

Input [Amplitude|Frequency|Phase]	Open Loop	Closed Loop	Containment
A, B, C: [±1500 N|1130 Hz|0]	E_A,B,C_: ±12.28 μm	E_A,B,C_: ±1.23 μm	A,B,C: 89.98%
A: [±1500 N|1130 Hz|π/2]	E_A_: ±31.48 μm	E_A_: ±1.32 μm	A: 95.81%
B: [±1500 N|1130 Hz|π]	E_B_: ±52.46 μm	E_B_: ±1.36 μm	B: 97.41%
C: [±1500 N|1130 Hz|0]	E_C_: ±44.71 μm	E_C_: ±1.37 μm	C: 96.93%
